# Genome-Wide Association Study Dissects Resistance Loci against Bacterial Blight in a Diverse Rice Panel from the 3000 Rice Genomes Project

**DOI:** 10.1186/s12284-021-00462-3

**Published:** 2021-02-27

**Authors:** Jialing Lu, Chunchao Wang, Dan Zeng, Jianmin Li, Xiaorong Shi, Yingyao Shi, Yongli Zhou

**Affiliations:** 1 National Key Facility for Crop Gene Resources and Genetic Improvement, Institute of Crop Sciences, Chinese Academy of Agricultural Sciences, Beijing, 100081 China; 2 College of Agronomy and Biotechnology, China Agricultural University, Beijing, 100193 China; 3 College of Agronomy, Anhui Agricultural University, Hefei, 230036 China

**Keywords:** Rice, Germplasm, GWAS, Bacterial blight

## Abstract

**Background:**

Bacterial blight (BB), caused by *Xanthomonas oryzae* pv. *oryzae* (*Xoo*) is one of the most devastating bacterial diseases of rice in temperate and tropical regions. Breeding and deployment of resistant cultivars carrying major resistance (*R*) genes has been the most effective approach for BB management. However, because of specific interaction of each *R* gene with the product of the corresponding pathogen avirulence or effector gene, new pathogen strains that can overcome the deployed resistance often emerge rapidly. To deal with ever-evolving *Xoo*, it is necessary to identify novel *R* genes and resistance quantitative trait loci (QTL).

**Results:**

BB resistance of a diverse panel of 340 accessions from the 3000 Rice Genomes Project (3 K RGP) was evaluated by artificial inoculation with four representative *Xoo* strains, namely Z173 (C4), GD1358 (C5), V from China and PXO339 (P9a) from Philippines. Using the 3 K RG 4.8mio filtered SNP Dataset, a total of 11 QTL associated with BB resistance on chromosomes 4, 5, 11 and 12 were identified through a genome-wide association study (GWAS). Among them, eight resistance loci, which were narrowed down to relatively small genomic intervals, coincided with previously reported QTL or *R* genes, e.g. *xa5*, *xa25*, *xa44(t)*. The other three QTL were putative novel loci associated with BB resistance. Linear regression analysis showed a dependence of BB lesion length on the number of favorable alleles, suggesting that pyramiding QTL using marker-assisted selection would be an effective approach for improving resistance. In addition, the Hap2 allele of *LOC_Os11g46250* underlying *qC5–11.1* was validated as positively regulating resistance against strain C5.

**Conclusions:**

Our findings provide valuable information for the genetic improvement of BB resistance and application of germplasm resources in rice breeding programs.

**Supplementary Information:**

The online version contains supplementary material available at 10.1186/s12284-021-00462-3.

## Background

Rice (*Oryza sativa* L.), an important cereal crop that supports more than 50% of the world population, suffers many biotic and abiotic stresses that lead to serious damage to crop yield and food safety (Oerke 2006). Rice bacterial blight (BB), caused by *Xanthomonas oryzae* pv. *oryzae* (*Xoo*), is one of the most destructive bacterial diseases worldwide, in both tropical and temperate regions (Mew 1978). As it is a vascular disease, chemical measures for controlling BB are less effective than for some other diseases (Niño-Liu et al. 2006). Breeding resistant cultivars is considered the most effective and eco-friendly strategy for managing this disease (McDonald and Linde 2002). Because of the adaptation between rice and *Xoo*, it is necessary to identify major resistance (*R*) genes and quantitative trait loci (QTL) that will support the development of new resistant varieties.

To date, 45 major resistance genes (denoted *Xa1* – *xa-45(t)*) that confer resistance to BB have been identified, and are distributed on all chromosomes except chromosomes 9 and 10 (Chukwu et al. 2019; Kim 2018; Kim and Reinke 2019; Neelam et al. 2020). Most BB resistance genes were detected through bi-parental population (F_2_ or recombinant inbred line populations) studies except for *Xa43(t)*, which was discovered in a multi-parent advanced generation inter-cross (MAGIC) population through genome-wide association study (GWAS) (Kim and Reinke 2019). Among the 45 major resistance genes, 11 genes (*Xa1*, *Xa3*/*Xa26*, *Xa4*, *xa5*, *Xa10*, *xa13*, *Xa21*, *Xa23*, *xa25*, *Xa27* and *xa41(t)*) have been cloned and characterized (https://shigen.nig.ac.jp/rice/oryzabase/). Because of either their lower level of resistance or their narrow spectrum of resistance, only a few genes (such as *Xa4*, *Xa21*, *Xa23* and *Xa39*) with broad-spectrum resistance have been widely deployed in breeding programs so far (Hu et al. 2017; Song et al. 1997; Wang et al. 2015; Zhang et al. 2015). Some resistance genes linked to undesirable agricultural traits cannot be directly applied in breeding, such as *xa13*, which improves BB resistance but reduces pollen fertility and seed setting rate (Li et al. 2020). Furthermore, with the emergence of new and more virulent *Xoo* strains, single resistance genes can be overcome in a short period (McDonald and Linde 2002). Hence, in order to manage this disease effectively, it is necessary to identify QTL associated with BB resistance using new strategies, and to pyramid multiple genes/QTL or introduce genes conferring broad-spectrum resistance and so breed durable resistance cultivars through advanced breeding programs.

Much progress has been made in identifying genes associated with desirable traits in rice using recent advanced sequencing technology (Peng et al. 2020; Wang et al. 2019; Wang et al. 2018). A large body of genotype data has been obtained in rice, including single nucleotide polymorphisms (SNPs), insertions and deletions (INDEL), structure variation (SV), and transposons, and these high-density markers have facilitated gene identification (Carpentier et al. 2019; Fuentes et al. 2019; Wang et al. 2020). In the past 5 years, GWAS based on SNP genotypes has become a popular and powerful tool to mine genes/QTL for complex agricultural traits including resistance to biotic and abiotic stress in rice, wheat and maize (Huang et al. 2015; Li et al. 2019b; Liu et al. 2017; Wang et al. 2016; Yano et al. 2019; Zhang et al. 2017b; Zhao et al. 2018). Different from traditional genetic mapping using bi-parental populations, GWAS can result in a relatively high mapping resolution and can detect more alleles at one locus by exploiting larger numbers of historical recombination events in varieties with more genetic diversity (Takeda and Matsuoka 2008). Notably, several genes related to rice diseases have been detected by GWAS (Kang et al. 2016; Zhang et al. 2017a; Zhang et al. 2019). For example, 13 resistance loci against rice black-streaked dwarf virus disease were detected in a rice diversity panel of 420 accessions using a 44 K SNP assay (Feng et al. 2019). Using the same set of germplasm and genotyping data, blast resistance was also evaluated by genome-wide association mapping and a new *Pik* allele was identified (Li et al. 2019a).

There have been several studies aimed at detecting rice BB resistance through GWAS. A MAGIC population of 1328 lines was evaluated for BB resistance, and QTL on chromosome 11 and 5 flanking known genes *Xa4* and *xa5* were discovered (Bandillo et al. 2013). Using a MAGIC population derived from eight parents, four QTL for Chinese weak virulent strain C2 and four QTL for strong virulent strain V were identified; two of them conferred resistance to both C2 and V (Chen et al. 2016). Two major loci, *qBLB11.1* and *qBLB5.1*, were identified for BB resistance in a rice MAGIC-plus population (Descalsota et al. 2018). The gene *Xa43(t)* was identified from a *japonica* MAGIC population of 120 lines and confirmed using a bi-parental population (Kim and Reinke 2019). A rice panel of 285 cultivars was inoculated with nine representative *Xoo* strains from Philippines to identify loci associated with BB resistance: strong associations were found for novel SNPs linked with known genes *Xa4*, *xa5*, *Xa7*, *xa13*, *Xa14*, *Xa21*, *xa25* and significant SNPs on chromosomes 6, 9, 11 and 12 were considered as novel sources of resistance (Dilla-Ermita et al. 2017). In addition, 12 genomic regions significantly associated with BB resistance against Philippines *Xoo* strains PXO61 (P1), PXO99 (P6), PXO339 (P9a) were identified in 172 *indica* rice accessions through GWAS, but no significant SNP for Chinese *Xoo* strains GD1358 (C5) or V was detected (Zhang et al. 2017a). Using 267 rice accessions, 15 QTL were identified for resistance against *Xoo* race C1 (Li et al. 2018). Eleven broad-spectrum resistance QTL were detected as effective against BB and bacterial leaf streak (Bossa-Castro et al. 2018). Also, by analyzing sequences of 1479 rice accessions, some selected regions were identified as overlapping with related agronomic trait genes *Rf1* and *SD1* and with BB resistance genes *Xa4* and *Xa26* (Xie et al. 2015).

BB is an important disease of rice and often occurs in Central and South China, resulting in heavy yield losses (Zhang 2009). Hence, it is imperative to exploit genetic resources and discover genes conferring resistance to representative *Xoo* strains from China. In this study, a diverse rice panel of 340 accessions from the 3000 Rice Genomes Project (3 K RGP) (Wang et al. 2018) was inoculated with three *Xoo* representative strains of epidemic pathotypes from China and a representative strain from Philippines to evaluate their resistance against BB. Based on the 3 K RG 4.8mio filtered SNP Dataset (Mansueto et al. 2017), QTL associated with BB resistance were identified by GWAS. The cultivars carrying high resistance to multiple strains and 11 QTL for BB resistance were identified. An uncharacterized gene *LOC_Os11g46250* was shown to be associated with resistance to strain C5. Our findings will facilitate the introduction of enhanced BB resistance in rice breeding programs.

## Methods

### Plant Materials and Evaluation of BB Resistance with Artificial Inoculation

A panel of 340 rice accessions was randomly selected from the sequenced accessions of the 3 K RGP, comprising 226 XI (*Xian*/Indica), 65 GJ (*Geng*/Japonica), 30 cA (circum-Aus), 8 cB (circum-Basmati) and 11 Admix (accessions between XI and GJ groups) (Wang et al. 2018). Seeds were soaked in sterilizing agent before sowing to prevent seed borne-disease and germinated in seedling trays with nutritional soil in the nursery house (25 °C, light 12 h/dark 12 h). The 30-day-old seedlings were transplanted to the experimental farm at the Institute of Crop Sciences, Chinese Academy of Agricultural Sciences, Beijing, China. All the accessions were grown by two rows in a randomized complete block design with three replications. Each row contained eight plants spaced 20 cm apart. The fertilizer for the experimental farm was applied according to the previous report (Zhai et al. 2020). During the plants growing period, the average temperature was about 28 °C and the daylength was 12–14 h in summer in Beijing. Agronomic management was performed according to local practices without bactericide.

The representative *Xoo* strains Z173 (C4), GD1358 (C5), and V from China were used to evaluate the resistance of the 340 rice accessions. The representative strain of Philippine race 9 PXO339 (P9a), which was used in different panels for GWAS in previous studies (Dilla-Ermita et al. 2017; Zhang et al. 2017a), was also used to identify novel resistance loci in this study. The four strains were cultured on potato peptone sucrose medium at 28 °C for 48 h (Zhou and Zhang 1999), and each inoculum was prepared by suspending the bacterial mass in sterile water at a concentration of 10^8^ cells ml^− 1^.

Four plants of each accession were inoculated with each strain in each replication using a leaf-clipping method (Kauffman et al. 1973) at the tillering stage (about 60-day-old seedlings). The lesion length (LL) was measured on the five uppermost leaves of each plant at 3 weeks post-inoculation when lesions were obvious and stable. The LL for each accession was calculated from four individual plants for each replication. The mean values of three replications for 340 rice accessions against each strain were used to generate a phenotype data matrix. Based on LL, the accessions were rated as resistant (LL < 5 cm), moderately resistant (5 cm ≤ LL < 10 cm), moderately susceptible (10 cm ≤ LL < 15 cm) and susceptible (LL ≥ 15 cm) (Zhang et al. 2017a).

### Genotype and Population Structure of Rice Accessions

The genotypes of 340 accessions were derived from the 3 K RG 4.8mio filtered SNP Dataset (Mansueto et al. 2017; http://snp-seek.irri.org/download.zul). Using the linkage disequilibrium (LD) pruning tool of PLINK 1.9 (Purcell et al. 2007), we obtained independent SNPs with genotype missing rate ≤ 5% and minor allele frequency ≥ 1% according to the settings “indep-pairwise 50 10 0.5”. These SNPs were used to construct a phylogenetic tree with PHYLIP v3.696 and iTOL v5 (Letunic and Bork 2006; Shimada and Nishida 2017). The genetic structure of all accessions was predicted with the ADMIXTURE program (Alexander et al. 2009). K values were set from 2 to 9 and the minimum coefficient of variation (CV) error value appeared at K = 3. Smart PCA in the software EIGENSOFT was used to perform principal component analysis (PCA) for calculating the subpopulation number (Galinsky et al. 2016).

### Genome-Wide Association Analysis of BB Resistance

A total of 4,130,496 SNPs from 340 rice accessions with the criteria of genotype missing rate ≤ 5%, minor allele frequency ≥ 1%, were obtained for association analysis. The Balding–Nichols method was used to develop a kinship matrix (Balding and Nichols 1995). The software Efficient Mixed-Model Association eXpedited (EMMAX) was used to perform GWAS through SNP genotypes and phenotype matrixes under PCA and kinship as covariations (Kang et al. 2010). GEC software was used to calculate the effective number of independent markers and significant *P*-value threshold (Li et al. 2012). Manhattan and quantile-quantile plots were created with the R package CMplot (https://github.com/YinLiLin/R-CMplot).

### Identification of QTL Associated with BB Resistance and Prediction of Candidate Genes

According to the previous studies, a region containing more than two SNPs above the significant *P*-value threshold in one estimated LD block were clustered as one QTL associated with BB resistance (Li et al. 2018; Guo et al. 2020) and the SNP with minimum *P*-value within each QTL was considered as the lead SNP (Zhang et al. 2019). Those chromosomes containing more than two QTL were referred to as hotspot chromosomes. The continuous region closely linked to the lead SNP (r^2^ ≥ 0.6) was considered as the local LD interval (Yano et al. 2016), and the heatmap of LD block was drawn with the R package LDheatmap (Purcell et al. 2007; Shin et al. 2006). SNP effects within QTL were annotated from Effect of 29mio biallelic SNPs on Rice Genome Annotation Project rel 7 gene models (https://snp-seek.irri.org).

Haplotype analysis for candidate genes within each QTL was performed based on the significant SNPs and these genes can be classified into several major haplotypes (at least containing 10 accessions). Then combining with phenotype data, variations in LL explained by different haplotypes were analyzed by ANOVA. The genes that displayed significant LL differences among haplotypes were chosen for further research.

### Analysis of the Effect of Favorable Alleles on BB Resistance

Each SNP has two alleles differing by a single nucleotide variation. Alleles with negative effects leading to shorter LL (associated with a more resistant phenotype) are referred to as favorable alleles (Bossa-Castro et al. 2018; Liu et al. 2017). The lead SNPs in the resistance loci were used to count the number of favorable alleles for all accessions and their allelic effects were determined between favorable alleles and LL using the ggplot2 package (Wickham 2009).

### Function Validation of Candidate Gene *LOC_Os11g46250*

The genomic DNA fragment of *LOC_Os11g46250*, comprising about 2 kb of promoter region, the full genomic sequence and 1 kb downstream sequence, was amplified from accession Yunlu 102 (3K_ID CX355) with the resistant allele (Hap2) of *LOC_Os11g46250.* The amplification reaction was performed under the following condition: 95 °C 3 min; 95 °C 15 s, 60 °C 15 s, 72 °C 6 min, 35 cycles; 72 °C 5 min. The fragment was cloned into plant binary vector pCAMBIA1300 (Biovector NTCC Inc., Beijing, China) digested by *Bam*H I and *Hin*d III to generate a complementary construct with the native promoter. Then the complementary construct was introduced into *Agrobacterium tumefaciens* strain EHA105 and subsequently transferred to rice variety Nipponbare (Nishimura et al. 2006). Transgenic lines were identified using the hygromycin resistance gene and a set of primers for the vector and gene fragment. Expression level of *LOC_Os11g46250* in the transgenic lines and wild-type were tested in 20-μL reactions using the TransScript Two-Step RT-PCR SuperMix (Trans, Beijing, China) following the manufacturer’s protocol via an ABI Prism 7900 Sequence Detection System (Applied Biosystems, Shanghai, China). The rice *ubiquitin* gene (*LOC_Os03g13170*) was used as the internal control. All primers used in this study are listed in Table S1.

To evaluate their resistance to strain C5, all the T_2_ lines and wild-type Nipponbare were planted in the transgenic experiment base (the same growing condition with the experiment farm) at the Institute of Crop Sciences, Chinese Academy of Agricultural Sciences, Beijing, China and inoculated with C5.

## Results

### Population Structure of Rice Accessions

**Fig. 1:**
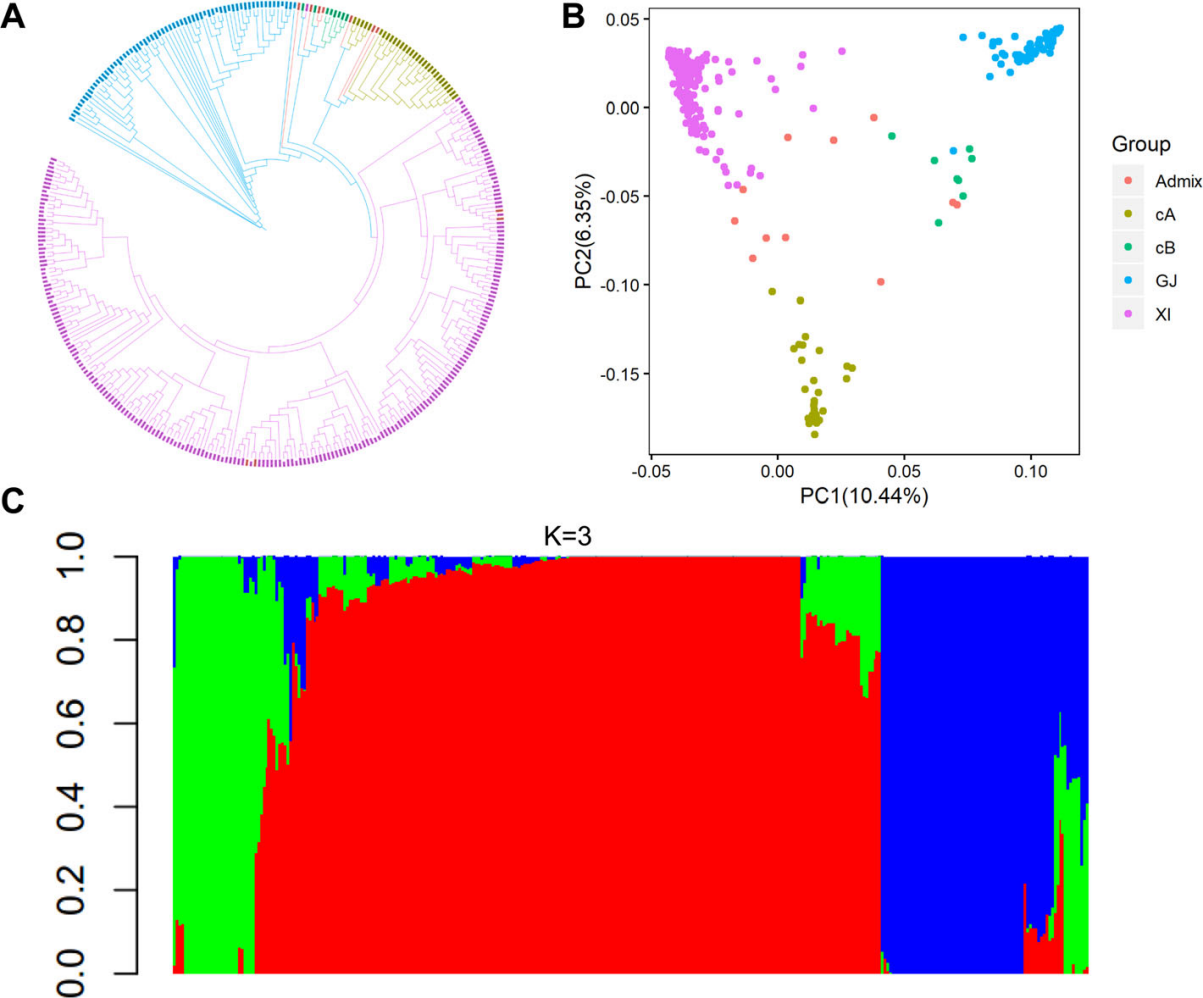
Population structure analysis of the diverse rice panel used in this study. **a** Phylogenetic tree of 340 rice accessions. **b** Principal component analysis for the first two components of 340 accessions. **c** Distribution of the estimated subpopulations component for individual accessions

Based on 527,165 independent SNPs with genotype missing rate ≤ 5% and minor allele frequency ≥ 1%, a phylogenetic tree was developed using the neighbor-joining method, dividing 340 accessions into four distinct major clusters (except for a small subgroup Admix), namely XI, GJ, cA and cB subgroups (Fig. 1a). Through principal component analysis, a similar result was observed, in which most of the genetic variation could be explained by the first two principal factors, largely consistent with the 3 K accessions classification (Fig. 1b). Based on the minimum CV error value (K = 3), all the accessions were grouped into three major subgroups (Fig. 1c). These results suggested that the accessions used as a covariate within the GWAS model displayed a clear population structure.

### Evaluation of Resistance to Four *Xoo* Strains

The distribution of lesion length (LL) in 340 accessions inoculated with four *Xoo* strains (C4, C5, V and P9a) showed large phenotypic variation (Fig. S1). Among the inoculated cultivars, eight accessions were highly resistant to all four strains with LL < 5 cm, and 54 were highly susceptible to all four strains with LL ≥ 15 cm (highlighted in green and yellow, respectively, in Table S2). Another cultivar IRBB 7 (3K_ID CX134) from Philippines conferred high resistance to three Chinese strains (LL 0.5–1.5 cm), but exhibited a loss of resistance to Philippines strain P9a (LL 15.8 cm). Of the cultivars that were highly susceptible to all strains, Chinese cultivars accounted for about 50%, suggesting that it is urgent to identify novel genes to deploy in rice breeding for China.

**Fig. 2:**
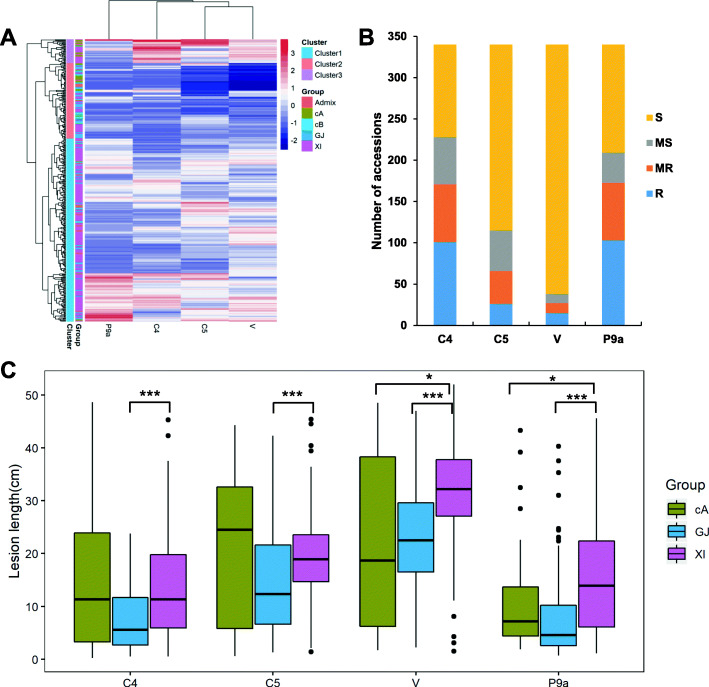
Bacterial blight resistance evaluation of 340 rice accessions inoculated with representative *Xoo* strains from China and Philippines. **a** Hierarchical cluster of accessions and strains based on lesion length (LL). **b** Number of accessions showing different reactions to four *Xoo* strains. R (LL < 5 cm), MR (5 cm ≤ LL < 10 cm), MS (10 cm ≤ LL < 15 cm), S (LL ≥ 15 cm). **c** Boxplots for LL of four *Xoo* strains in three major subgroups. Box edges represent the 0.25 and 0.75 quantiles with median values indicated by bold lines. * and *** denote significant differences in mean LL among subpopulations at *P* < 0.05, 0.001, respectively

Based on the LL of all accessions, the four *Xoo* strains were divided into two obvious groups, namely Philippines strain group (P9a) and Chinese strain group (C4, C5 and V) (Fig. 2a). At the same time, 340 rice accessions formed three major clusters, based on the reaction to the four strains. Cultivars within one cluster exhibited similar resistance against different strains (Fig. 2a). According to classification by LL, a large number of accessions were moderately susceptible or susceptible, with 49.71%, 80.59%, 92.06% and 49.12% for strains C4, C5, V and P9a, respectively (Fig. 2b). For all strains, the average LL of subgroup GJ was significantly shorter than that of subgroup XI (*P* < 0.001). Also, for both strains V and P9a, the average LL of subgroup XI was significantly longer than that of subgroup cA (*P* < 0.05) (Fig. 2c). These results indicated that rice cultivars in subgroup XI were more susceptible than those in other subgroups.

### Identification of Resistance Loci against BB

**Fig. 3:**
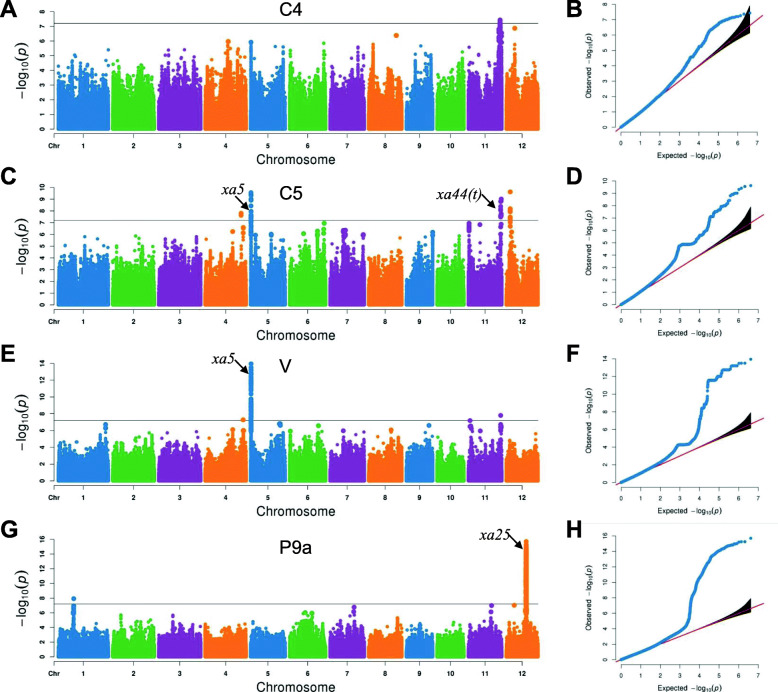
Genome-wide association study of rice resistance to four *Xoo* strains. **a**, **c**, **e**, **g** Manhattan plots of GWAS results for strains C4, C5, V, and P9a, respectively. **b**, **d**, **f**, **h** Quantile-quantile plots of expected and observed −log_10_ (*P*-value) for strains C4, C5, V, and P9a, respectively. Horizontal black line indicates the significant *P*-value threshold of 6.31E− 8. The arrows indicate the reported bacterial blight resistance genes

To dissect genome-wide associated resistance loci for three Chinese *Xoo* strains and one Philippines *Xoo* strain, we performed GWAS with a mixed linear model of the EMMAX program using 4,130,496 high quality SNPs and LL as genotype and phenotype data, respectively. Based on the effective number of independent markers, the threshold of significant *P*-value was estimated to be 6.31E− 8 by the Bonferroni correction method. In total, we identified 11 QTL within 1576 unique SNPs associated with BB resistance to four strains (Fig. 3; Table 1). Among them, one, five, two and three QTL were detected to be associated with resistance to strains C4, C5, V and P9a, respectively. In detail, these significant SNPs were distributed on chromosomes 4, 5, 11 and 12, including four significant SNPs for C4, 80 for C5, 370 for V and 1173 for P9a (Table S3). Interestingly, 51 significant SNPs were identified as associated with resistance to both C5 and V, suggesting that resistance to distinct strains might be controlled by the same resistance loci.

**Table 1:** Significant loci for rice bacterial blight resistance obtained from genome-wide association study

QTL name^**a**^	LD block interval (bp)	Number of significant SNP	Lead SNP position (bp)	Lead SNP ***P***-value	Overlapped with known genes/QTL
*qC4–11*	27,382,744–27,579,384	4	27,490,677	3.70E-08	L11(Zhang et al. 2017a), *QBbr11–1*(Chen et al. 2016)
*qC5–4*	31,060,503–31,170,315	2	31,071,832	1.60E-08	novel
*qC5–5*	283,658–476,382	51	348,262	2.81E-10	*xa5*(Iyer-Pascuzzi et al. 2008)
*qC5–11.1*	27,985,691–28,013,096	8	27,996,768	1.58E-09	*xa44(t)* (Kim 2018), L11(Zhang et al. 2017a), *QBbr11–2*(Chen et al. 2016)
*qC5–11.2*	28,430,697–28,479,727	7	28,479,727	9.75E-10	L11(Zhang et al. 2017a), *QBbr11–2*(Chen et al. 2016)
*qC5–12*	3,460,540–3,549,818	12	3,493,693	2.40E-10	novel
*qV-5.1*	288,855–443,952	272	436,664	1.14E-14	*xa5*(Iyer-Pascuzzi et al. 2008)
*qV-5.2*	444,857–476,382	98	464,676	3.55E-10	novel
*qP9a-12.1*	17,003,758–17,085,897	65	17,016,036	1.58E-13	L12(Zhang et al. 2017a)
*qP9a-12.2*	17,169,665–17,339,744	498	17,274,286	2.03E-16	L12(Zhang et al. 2017a), *xa25*(Liu et al. 2011)
*qP9a-12.3*	17,342,010–17,426,793	610	17,402,252	1.03E-14	L12(Zhang et al. 2017a)

^a^*qX-N* indicates QTL located on chromosome N conferring resistance to X

For strain C4, we identified only one resistance locus, *qC4–11*, defined by four significant SNPs within the region 27,382,744–27,579,384 bp on chromosome 11. These four SNPs were located in *OsGPAT3* (*LOC_Os11g45400*), which played an indispensable role in pollen and anther development, including anther wall programmed cell death (Men et al. 2017; Sun et al. 2018).

For strain C5, five QTL were identified as associated with BB resistance, located on chromosomes 4, 5, 11 (two QTL), and 12. Notably, a cluster of significant SNPs on chromosome 11 were located in the genomic region 27,985,691–28,479,727 bp. Based on linkage disequilibrium analysis, the continuous region of chromosome 11 were divided into two QTL, namely *qC5–11.1* and *qC5–11.2*.

For strain V, there was a steep peak of resistance-related SNPs on chromosome 5, defined by two QTL. The *qV-5.1* region harbored 272 significant SNPs, overlapping with a broadly effective recessive resistance gene *xa5*. Notably, no QTL or gene for BB resistance was previously reported in the other QTL, *qV-5.2* (444,857–476,382 bp), spanning about 31 kb on chromosome 5. This region contained 98 significant SNPs and might include potential novel BB genes.

For strain P9a, we identified 1173 significant SNPs associated with BB resistance on chromosome 12. Based on linkage disequilibrium analysis, this region was divided into three QTL (*qP9a-12.1*–*qP9a-12.3*). Among them, *qP9a-12.2* was located close to the reported race-specific resistance gene *xa25*.

### Hotspot Chromosomes Associated with BB Resistance

The above GWAS results indicated chromosomes 5, 11, and 12 as hotspots for BB resistance. Strikingly, chromosome 11 contained multiple resistance loci for different *Xoo* strains, including one for C4 and two for C5, i.e. *qC4*–*11*, *qC5*–*11.1* and *qC5*–*11.2* (Table 1). The sole QTL related to C4 resistance, *qC4–11*, contained about 196 kb interval, in which the significant SNPs were located in the region of *OsGPAT3*. Of the QTL for C5 resistance, *qC5–11.1* spanned an approximately 27 kb interval (27,985,691–28,013,096 bp), while *qC5–11.2* spanned about 49 kb. Of particular note, a recessive gene *xa44(t)*, conferring resistance to *Xoo* isolate HB1009 (K3a) from Korea, was exactly located in the interval of *qC5–11.1* (Kim 2018), but no significant SNP was detected within the candidate genes of *xa44(t)*, *Os11g0690466* and *Os11g0690066*. Using the 3 K RG 4.8mio filtered SNP Dataset, we found no SNP variation for *Os11g0690466* among 340 accessions, while 120 SNPs for *Os11g0690066* clustered the accessions into three major haplotypes (Table S4). ANOVA showed no significant difference in LL among three major haplotypes for any *Xoo* strains used in this study (Fig. S2). Furthermore, we analyzed the expression of *xa44(t)* in the resistant and susceptible cultivars, and found no difference between them, suggesting that the SNP variation and expression of *xa44(t)* were not responsible for *qC5*–*11.1* and other genes responsible for *qC5*–*11.1* should be studied.

Chromosome 5, as the second hotspot chromosome, contained one QTL against strain C5 and two QTL against strain V. Both *qC5–5* and *qV-5.1* overlapped with *LOC_Os05g01710* (*xa5*), a recessive gene encoding transcription initiation factor IIA subunit 2, responsible for broad-spectrum resistance (Huang et al. 2016; Iyer-Pascuzzi et al. 2008; Mishra et al. 2013). Haplotype analysis of *LOC_Os05g01710*, based on significant SNPs, showed that accessions carrying Hap2 were more resistant to four strains than those with Hap1 (Fig. S3A, S3B), and the Hap2 allele was authentically *xa5.* Accessions carrying the Hap2 allele belonged to the cA subgroup, suggesting that cA subgroup could be used as an important resistance source in future breeding programs. Notably, *qV-5.2* was not reported to be associated with BB resistance in previous studies (Table 1), and might be a novel BB resistance locus. The lead SNP of *qV-5.2* (rs5_464,676, *P-*value = 3.55E− 10) was located in the vicinity of transposon gene *LOC_Os05g01770* (Table S3), suggesting that this transposon gene might confer resistance to strain V.

Chromosome 12, as the third hotspot chromosome, carried the largest number of QTL, including one QTL for resistance to strain C5 and three for P9a, but no common QTL was detected for resistance to both strains, indicating that they were controlled by different genes. There were no reported BB resistance genes in the region of *qC5–12*. The significant SNP rs12_3471439 within *qC5–12* contributed to a stop codon gained for a transposon gene (*LOC_Os12g07080*), potentially the causal gene conferring resistance to C5. Three QTL for resistance to P9a were all located in the interval of L12 loci, in the range of 16,502,066–17,531,046 bp on chromosome 12, and were also reported to be associated with resistance to P9a (Zhang et al. 2017a). Among them, *qP9a-12.2* harbored a recessive and race-specific gene *xa25*, associated with resistance to P9a (Liu et al. 2011). Haplotype analysis of *xa25*, based on significant SNPs, divided the accessions into four major haplotypes (Fig. S4A). A significant difference in LL among different haplotypes was observed only for P9a, confirming the race-specific resistance of *xa25* (Fig. S4B).

### The Effect of Pyramiding Favorable Alleles on BB Resistance

To further explore the comprehensive effects of different alleles on the reaction to BB, the number of favorable alleles in each accession were examined. For each QTL, we selected the lead SNP to represent the QTL effect. In general, each accession contained zero to four favorable alleles. Through linear regression analysis, significant correlations were observed between LL and number of favorable allele with *R*^2^ = 0.88, 0.71, 0.89 for C5, V and P9a, respectively (Fig. S5). For *Xoo* strains C5 and P9a, the LL of the accessions significantly decreased as the number of favorable allele increased (Fig. S5A, S5C). For V, there was a slight difference between LL and the number of favorable allele (Fig. S5B) and we speculated that this effect might involve the influence of genetic background. In general, the LL of cultivars with multiple favorable alleles was shorter than those with a single allele, but their genetic background should be considered when pyramiding favorable alleles in breeding programs.

### *LOC_Os11g46250* Positively Regulates Resistance to C5

**Fig. 4:**
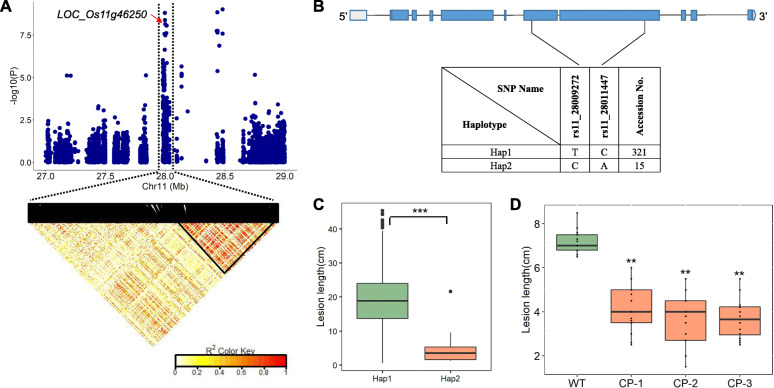
Analysis of associated region of *qC5*–*11.1* and its candidate gene *LOC_Os11g46250*. **a** Manhattan plot (top) and linkage disequilibrium heatmap (bottom) of genomic region surrounding *qC5*–*11.1*. The red arrow indicates the position of significant SNPs located in *LOC_Os11g46250.* **b** Exon-intron structure and haplotypes of *LOC_Os11g46250* derived from significant SNPs. Rectangles and lines represent exons and introns, respectively and the coding sequence highlighted in blue. **c** Lesion length (LL) of accessions with different haplotypes of *LOC_Os11g46250* inoculated with strain C5. **d** LL of transgenic lines and wild-type inoculated with C5. Box edges represent the 0.25 and 0.75 quantiles with median values indicated by bold lines. ** and *** refer to significant differences at *P* < 0.01 and 0.001, respectively. WT and CP-1/CP-2/CP-3 refer to wild-type Nipponbare and three independent transgenic lines carrying complementary genomic fragments, respectively

Since two candidate genes of *xa44(t)* were not responsible for *qC5–11.1*, we focused on other genes in this region. In the candidate region of *qC5*–*11.1* spanning 27,985,691–28,013,096 bp, two significant SNPs with *P-*value of 8.80E− 09 (rs11_28009272 and rs11_28011447) were exactly located within *LOC_Os11g46250*, which encodes an uncharacterized protein (Fig. 4a, b). Using these two significant SNPs to perform haplotype analysis, all the accessions were divided into two major haplotypes (Fig. 4b). Intriguingly, the average LL of the accessions carrying Hap2 was significantly shorter than those carrying Hap1 when inoculated with strain C5, indicating that this candidate gene was likely to be associated with BB resistance (Fig. 4c). Transgenic lines carrying a complementary genomic construct of *LOC_Os11g46250*, amplified from variety Yunlu 102 with the Hap2 allele of *LOC_Os11g46250* driven by its native promoter, were obtained. The qRT-PCR analysis revealed that Hap2 was expressed in all transgenic lines but not in the wild-type, while the expression level of Hap1 in the transgenic lines and wild-type had no significant difference (Fig. S6). When inoculated with C5 to evaluate BB resistance, the transgenic lines showed significantly shorter lesions compared to the wild-type, with LL decreased by about 50% at 3 weeks post-inoculation (Fig. 4d). These results demonstrated that *LOC_Os11g46250* was associated with resistance to Chinese virulent strain C5 and the Hap2 allele positively regulates resistance against C5.

## Discussion

Accurate genotyping is crucial to the success of any large-scale genetic association study (Tam et al. 2019). Compared to 44 K, 55 K and 700 K SNP arrays, SNPs identified from the 3 K RG 4.8mio filtered SNP Dataset (derived from the 3 K RGP) in this study were distributed more densely and covered almost all registered genes in the Nipponbare reference genome (Mansueto et al. 2017; McCouch et al. 2016; Meng et al. 2017; Zhao et al. 2011). The sequencing data of the 3 K RGP have provided a basis for identifying QTL associated with agronomic traits, abiotic resistance and sheath blight resistance in rice (Shi et al. 2017; Zhai et al. 2018; Zhang et al. 2017b; Zhang et al. 2019). In this study, QTL against BB in a diverse rice panel from the 3 K RGP were dissected through GWAS, and the results obtained provide novel information for rice breeding.

### Diverse Reactions to *Xoo* Strains among Different Accessions

We evaluated the resistance of 340 rice accessions from the 3 K RGP to BB caused by four *Xoo* strains. Strain PXO339 is a representative strain of Philippines race 9, and the other three strains were collected in rice growing regions of China. Strain Z173 (C4) is a representative strain of pathotype 4 from the Yangtze River basin (Fang et al. 1990). Strains GD1358 (C5) and V are new virulent strains that arose in Guangdong Province which are currently prevalent in the southern rice-growing regions of China; these two strains are virulent to *R* genes *Xa4* and *Xa21*, respectively (Zeng et al. 2002; Zhang 2005). In all, only eight high resistant cultivars with LL < 5 cm for all these four strains were identified, including two XI, two GJ, two cA and two Admix cultivars (Table S2). Only one Chinese cultivar, Yunlu 102 (3K_ID CX355), was highly resistant to all four strains. Besides that cultivar, accessions CX134, CX220, CX269 and IRIS_313–11,051 showed a high level of resistance to strains C5 and V, but only moderate resistance or resistance to C4 and P9a, except for CX134, which exhibited high susceptibility to P9a (Table S2). These sources of resistant germplasm in different genetic backgrounds can provide valuable material for facilitating breeding for BB resistance.

### Comparison with BB QTL and Genes Identified in Previous Studies

In previous studies, BB resistance QTL and candidate genes were identified from bi-parental populations, MAGIC populations and natural populations (Bandillo et al. 2013; Chen et al. 2016; Descalsota et al. 2018; Dilla-Ermita et al. 2017; Kim and Reinke 2019; Li et al. 2018; Xie et al. 2015; Zhang et al. 2017a), allowing for a comparison between loci in this study and previously reported QTL and genes. Through our use of high-density SNP markers, QTL identified in the present research were narrowed down to a small genomic region enabling cross-reference with other reported genes and QTL spanning a larger region.

Of the 11 QTL detected in this study, three QTL on chromosome 11 all exactly overlapped with L11, which was associated with resistance to strains P1 and P6 (Zhang et al. 2017a). *QBbr11–1* contained *qC4–11*, while *QBbr11–2* contained *qC5–11.1* and *qC5–11.2* (Chen et al. 2016). Notably, a recessive BB resistance gene *xa44(t)* was also located in the region of *qC5–11.1* (Kim 2018). Two QTL on chromosome 5, *qC5–5* and *qV-5.1*, were co-located with the broad-spectrum resistance gene *xa5* (Iyer-Pascuzzi et al. 2008). Three QTL on chromosome 12 overlapped with L12, which confers resistance to P9a, and *qP9a-12.2* coincided with *xa25* and a significant association signal for P9a and P9b on chromosome 12 in previous reports (Liu et al. 2011; Zhang et al. 2017a; Dilla-Ermita et al. 2017). As the loci were identified using different *Xoo* strains, it was possible that these regions conferred resistance to multiple strains or certain single genes conferred independent resistance and functioned together.

Besides these QTL overlapping with known resistance genes, three QTL (*qC5–4*, *qC5–12*, *qV-5.2*) were newly discovered in the present study, located a long distance on the physical map from known BB resistance QTL or genes (Table 1). To our knowledge, this is the first report of QTL associated with prevalent Chinese *Xoo* strains. In previous research, 172 *indica* accessions were inoculated with strains C5 and V, but no resistance loci were detected in that study (Zhang et al. 2017a). The significant SNP rs4_31,060,503 within *qC5–4* was located in the promoter of *OsHyPRP16* (*LOC_Os04g52260*), which contained 28 *cis*-regulatory elements (14 WRKY71OS, 6 WBOXATNPR1, 1 GT1GMSCAM4, 1 WBBOXPCWRKY1, 6 BIHD1OS) that are involved in pathogen, elicitors and disease resistance response (Kapoor et al. 2019). So it might be important to establish whether *OsHyPRP16* is responsible for resistance to C5. Also, one significant SNP rs12_3471439, located within *qC5–12* caused a stop codon in the transposon gene *LOC_Os12g07080*, which might account for the resistance to C5. In the *qV-5.2* interval, four significant SNPs of *LOC_Os05g01760*, encoding putative lysine ketoglutarate reductase trans-splicing related 1, formed two major haplotypes (Table S3; Fig. S7A). The lesions of accessions with Hap2 were significantly shorter than those with Hap1 for strain V and the other three strains, suggesting that the causal gene underlying the QTL might confer broad-spectrum resistance (Fig. S7B). Thus, these novel QTL and known QTL/genes provide new insights for breeding high-resistant rice cultivars and elucidating the interaction mechanism between *Xoo* and rice.

Large numbers of transposable elements exist in rice genomes (Carpentier et al. 2019; Liu et al. 2020). Currently, significant progress has been made in understanding the molecular mechanisms of transposon-mediated development and defense in plants. For example, some transposon-derived DNA binding proteins, transcription factors and transposases identified in animals, fungi and plants function as developmental regulators in diverse pathways (Bundock and Hooykaas 2005; Feschotte 2008; Robertson 2002). Transposon-derived small RNA TE-siR815 were involved in BB resistance (Zhang et al. 2016). Strikingly, we found 376 significant SNPs associated with BB resistance located in or in the vicinity of five transposons and 17 retrotransposons (Table S3). Several transposons and retrotransposon were also anchored by significant SNPs associated with BB and sheath blight resistance in previous studies (Zhang et al. 2017a; Zhang et al. 2019). However, less is known about the effect of SNPs in transposon on its function. It is necessary to further exploit the mechanism of transposon involved in regulating BB resistance in the future.

### Potential Application of QTL in Rice Breeding

It is known that chromosome 11 is an important and complex region of the rice genome with respect to BB resistance, containing mapped or finely-mapped BB *R* genes *Xa22(t)*, *Xa30(t)*, *Xa32(t)*, *Xa35(t)*, *Xa36(t)*, *Xa39*, *Xa40*, *xa41(t)*, *Xa43(t)* and *xa44(t)*, and cloned genes *Xa3*/*Xa26*, *Xa4*, *Xa10*, *Xa21*, *Xa23* (https://shigen.nig.ac.jp/rice/oryzabase/). Because of the wide deployment of single *R* genes and *Xoo*-rice coevolution, elite *R* genes *Xa4* and *Xa21* have been overcome by the newly-emerged *Xoo* strains C5 and V in China (Zeng et al. 2002; Zhang 2005). In this study, we identified a novel gene *LOC_Os11g46250* within *qC5–11.1* that was responsible for positively regulating C5 resistance (Fig. 4), providing a new gene for rice breeding and enriching our knowledge of BB *R* genes on chromosome 11.

Furthermore, chromosome 5 also contains resistance loci for strains C5 and V. Notably, *qC5–5* and *qV-5.1* partially overlapped, and a broad-spectrum resistance gene *xa5* was found to be located exactly in that region (Iyer and McCouch 2004). Based on haplotype analysis and LL, we found accessions carrying Hap2 (*xa5*) showed greater resistance to strains C4, C5, V and P9a than those carrying Hap1 (Fig. S3). Also, cultivar IRBB5 carrying *xa5* exhibited significantly shorter LL for strains P1, PXO341 (P10), OS198 (C6) and IV than IR24, the recurrent parent of IRBB5 (data unpublished), indicating that *xa5* conferred pleiotropic resistance to *Xoo* strains. As most accessions contain the susceptible allele of *xa5*, it is likely to improve BB resistance by editing this gene using the CRISPR/Cas9 technique (Ma and Liu 2016). However, a few *Xoo* strains containing *pthXo1*, such as PXO99A and PXO71, were compatible with rice plants with *xa5* (Huang et al. 2016), suggesting that *xa5* should be combined with other genes/QTL to prevent more virulent strains spreading. To this end, *xa5* has already been combined with *Xa4*, *Xa7*, *xa13* and *Xa21* in other breeding programs (Pradhan et al. 2015; Hsu et al. 2020). Therefore, pyramiding *xa5* and other QTL can be an effective and eco-friendly approach for improving BB resistance.

## Conclusion

In the present study, GWAS for bacterial blight resistance was performed with a diverse panel of 340 rice accessions using the 3 K RG 4.8mio filtered SNP Dataset and their phenotypes after inoculating with three Chinese *Xoo* strains and one Philippines strain. Eight known and three novel resistance loci were identified and several candidate genes were predicted through gene annotation and haplotype analysis. Moreover, an uncharacterized gene *LOC_Os11g46250* underlying *qC5–11.1* was validated as associated with resistance to C5 and the allele of Hap2 positively regulated resistance against C5. Building on our useful new SNP information, QTL pyramiding using marker-assisted selection would be an effective approach for improving BB resistance in rice.

## Supplementary Information

**Additional file 1: Table S1.** Primers used in this study

**Additional file 2: Table S2.** Information about the diverse rice panel of 340 accessions used in this study.

**Additional file 3: Table S3.** Annotation of significant SNPs for bacterial blight resistance in a diverse panel of 340 accessions

**Additional file 4: Table S4.** Haplotypes of one candidate gene of *xa44(t)*, *Os11g0690066*

**Additional file 5: Fig. S1.** Distribution of bacterial blight lesion length among 340 accessions. Box edges represent the 0.25 and 0.75 quantiles with median values indicated by bold lines. **Fig. S2.** Lesion length of accessions carrying different haplotypes of one candidate gene of *xa44(t)*, *Os11g0690066*. Characters above boxplots indicate significant differences according to Duncan’s multiple comparison tests (*P* < 0.05). **Fig. S3.** Haplotype analysis of *xa5*. (A) Exon-intron structure and haplotypes based on significant SNPs. Rectangles and lines represent exons and introns, respectively and the coding sequence highlighted in blue. (B) Lesion length of accessions with different haplotypes. Box edges represent the 0.25 and 0.75 quantiles with median values indicated by bold lines. Characters above boxplots indicate significant differences according to Duncan’s multiple comparison tests (*P* < 0.05). **Fig. S4.** Haplotype analysis of *xa25*. (A) Exon-intron structure and haplotypes based on significant SNPs. Rectangles and lines represent exons and introns, respectively and the coding sequence highlighted in blue. (B) Lesion length of accessions with different haplotypes. Box edges represent the 0.25 and 0.75 quantiles with median values indicated by bold lines. Characters above boxplots indicate significant differences according to Duncan’s multiple comparison tests (*P* < 0.05). **Fig. S5.** Linear regression analysis of the number of favorable alleles and lesion length of *Xoo* strains (A) C5, (B) V and (C) P9a. **Fig. S6.** Relative expression level of Hap1 (A) and Hap2 (B) of *LOC_Os11g46250* in transgenic lines and wild-type. *UBQ* denotes the rice* ubiquitin* gene (*LOC_Os03g13170*) as the internal control; ** refers to significant differences at *P* < 0.01; WT and CP-1/CP-2/CP-3 refer to wild-type Nipponbare and three independent transgenic lines carrying complementary genomic fragments, respectively. **Fig. S7.** Haplotype analysis of *LOC_Os05g01760* underlying *qV-5.2*. (A) Exon-intron structure and haplotypes based on significant SNPs. Rectangles and lines represent exons and introns, respectively and the coding sequence highlighted in blue. (B) Lesion length of accessions with different haplotypes. Box edges represent the 0.25 and 0.75 quantiles with median values indicated by bold lines. Characters above boxplots indicate significant differences according to Duncan’s multiple comparison tests (*P* < 0.05).

## Data Availability

The dataset supporting the conclusions of this article is provided within the article and its additional files.

## Abbreviations

BB: Bacterial blight
*Xoo*: *Xanthomonas oryzae* pv. *oryzae*
GWAS: Genome-wide association study
3 K RGP: 3000 Rice Genomes Project
LL: Lesion length
MAGIC: Multi-parent advanced generation inter-cross
