# Supplementary material for: Genome-Wide Association Study Dissects Resistance Loci against Bacterial Blight in a Diverse Rice Panel from the 3000 Rice Genomes Project
Source: Rice (N Y). 2021 Feb 27;14:22. doi: 10.1186/s12284-021-00462-3 (PMC7914325; doi:10.1186/s12284-021-00462-3)
Supplement: Supplementary file 5 — Additional file 5: Fig. S1. Distribution of bacterial blight lesion length among 340 accessions. Box edges represent the 0.25 and 0.75 quantiles with median values indicated by bold lines. Fig. S2. Lesion length of accessions carrying different haplotypes of one candidate gene of xa44(t), Os11g0690066. Characters above boxplots indicate significant differences according to Duncan’s multiple comparison tests (P < 0.05). Fig. S3. Haplotype analysis of xa5. (A) Exon-intron structure and haplotypes based on significant SNPs. Rectangles and lines represent exons and introns, respectively and the coding sequence highlighted in blue. (B) Lesion length of accessions with different haplotypes. Box edges represent the 0.25 and 0.75 quantiles with median values indicated by bold lines. Characters above boxplots indicate significant differences according to Duncan’s multiple comparison tests (P < 0.05). Fig. S4. Haplotype analysis of xa25. (A) Exon-intron structure and haplotypes based on significant SNPs. Rectangles and lines represent exons and introns, respectively and the coding sequence highlighted in blue. (B) Lesion length of accessions with different haplotypes. Box edges represent the 0.25 and 0.75 quantiles with median values indicated by bold lines. Characters above boxplots indicate significant differences according to Duncan’s multiple comparison tests (P < 0.05). Fig. S5. Linear regression analysis of the number of favorable alleles and lesion length of Xoo strains (A) C5, (B) V and (C) P9a. Fig. S6. Relative expression level of Hap1 (A) and Hap2 (B) of LOC_Os11g46250 in transgenic lines and wild-type. UBQ denotes the rice ubiquitin gene (LOC_Os03g13170) as the internal control; ** refers to significant differences at P < 0.01; WT and CP-1/CP-2/CP-3 refer to wild-type Nipponbare and three independent transgenic lines carrying complementary genomic fragments, respectively. Fig. S7. Haplotype analysis of LOC_Os05g01760 underlying qV-5.2. (A) Exon-intron structure an [file 12284_2021_462_MOESM5_ESM.pptx]

## Slide 1
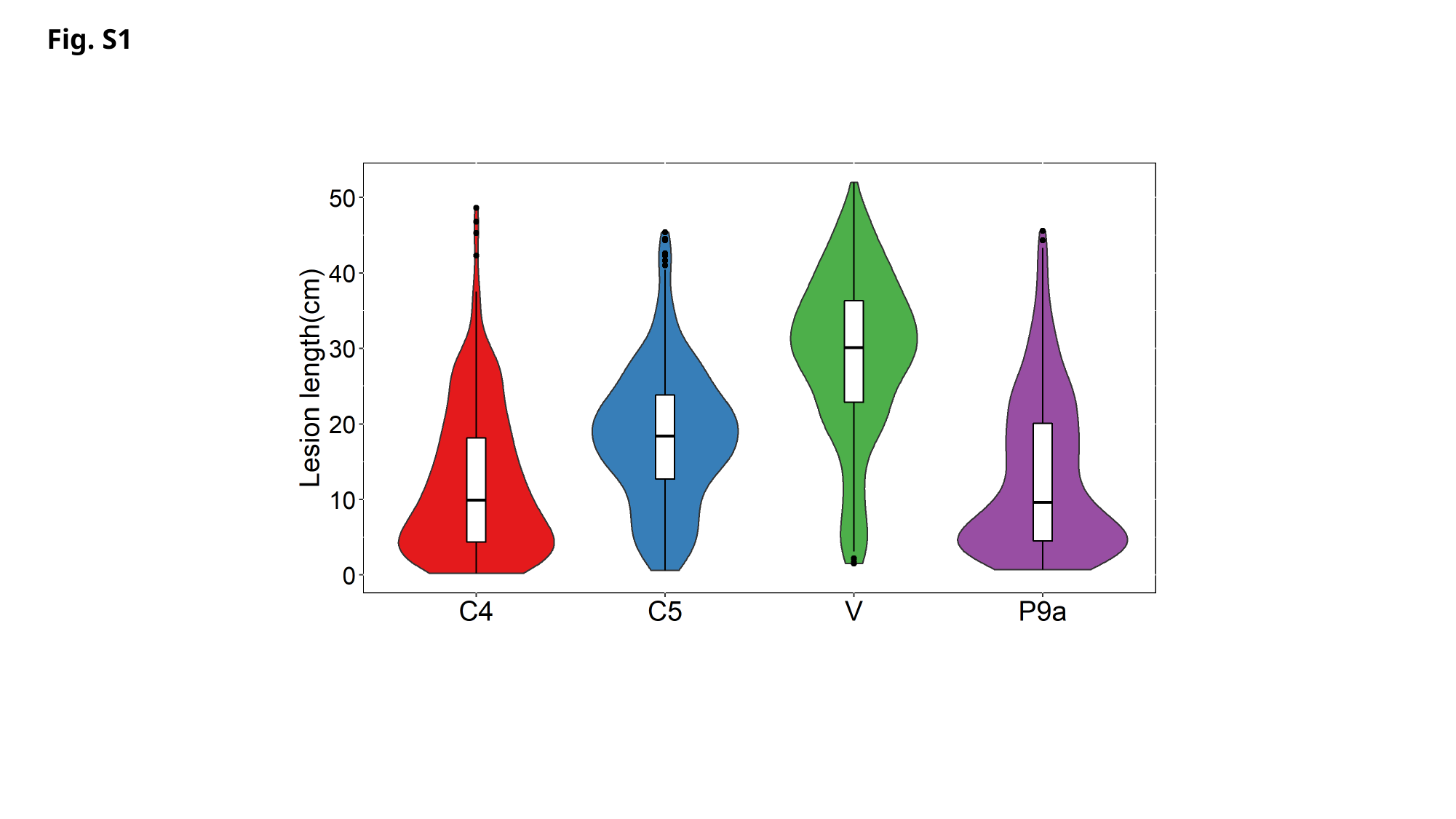

Fig. S1

## Slide 2
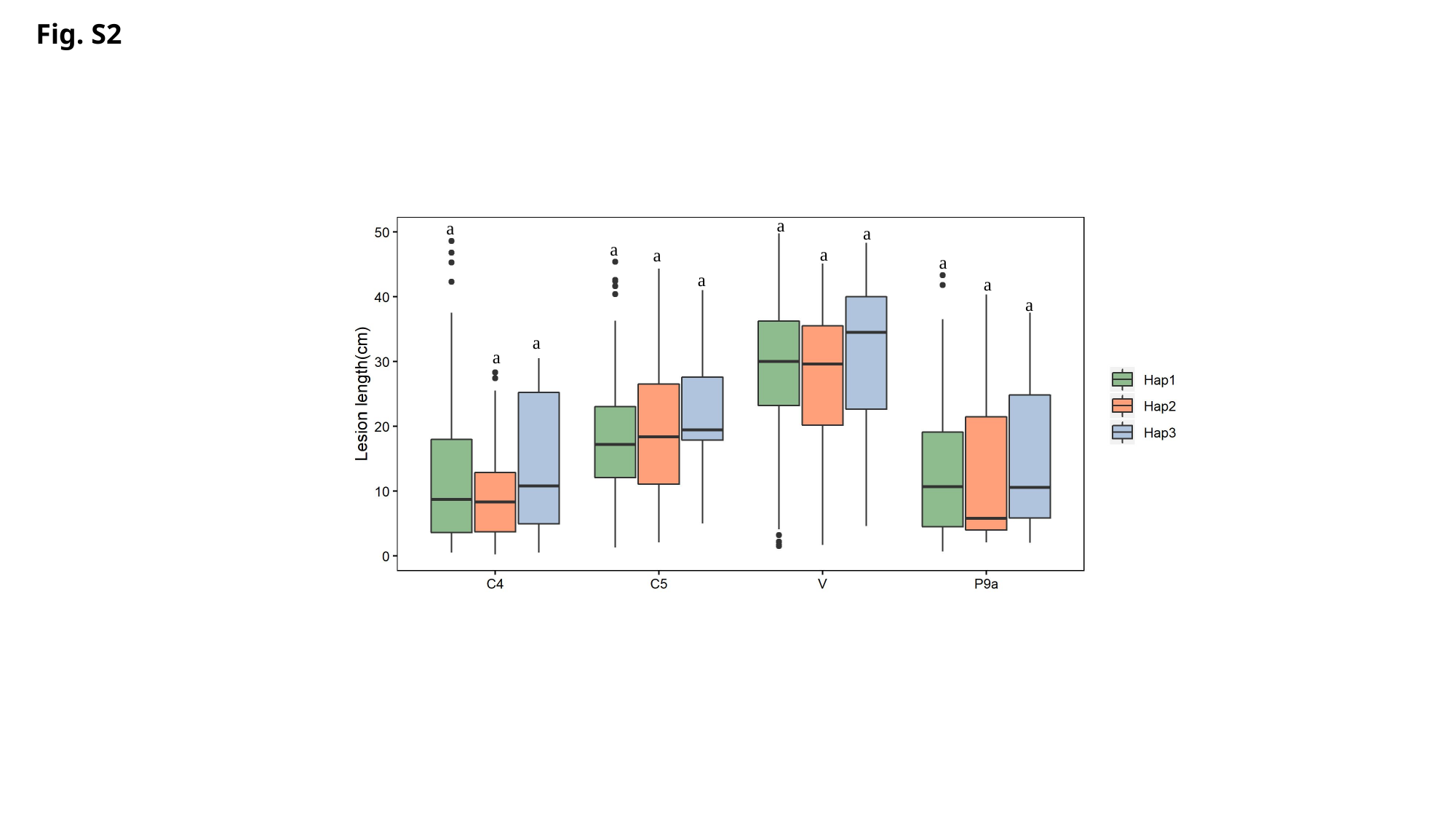

Fig. S2
a
a
a
a
a
a
a
a
a
a
a
a

## Slide 3
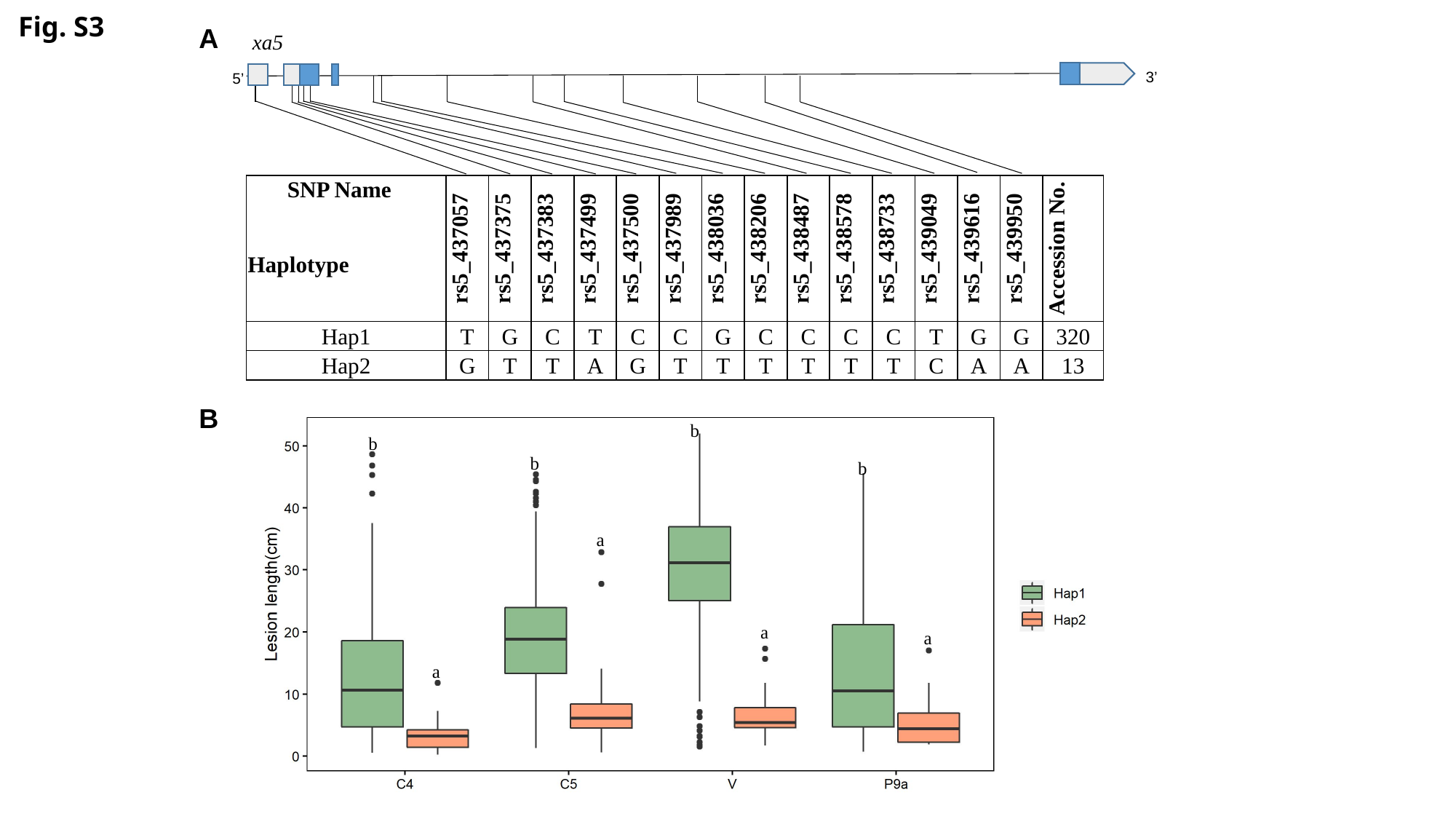

Fig. S3
A
xa5
3’
5’
| SNP Name Haplotype | rs5\_437057 | rs5\_437375 | rs5\_437383 | rs5\_437499 | rs5\_437500 | rs5\_437989 | rs5\_438036 | rs5\_438206 | rs5\_438487 | rs5\_438578 | rs5\_438733 | rs5\_439049 | rs5\_439616 | rs5\_439950 | Accession No. |
| --- | --- | --- | --- | --- | --- | --- | --- | --- | --- | --- | --- | --- | --- | --- | --- |
| Hap1 | T | G | C | T | C | C | G | C | C | C | C | T | G | G | 320 |
| Hap2 | G | T | T | A | G | T | T | T | T | T | T | C | A | A | 13 |
B
b
b
b
b
a
a
a
a

## Slide 4
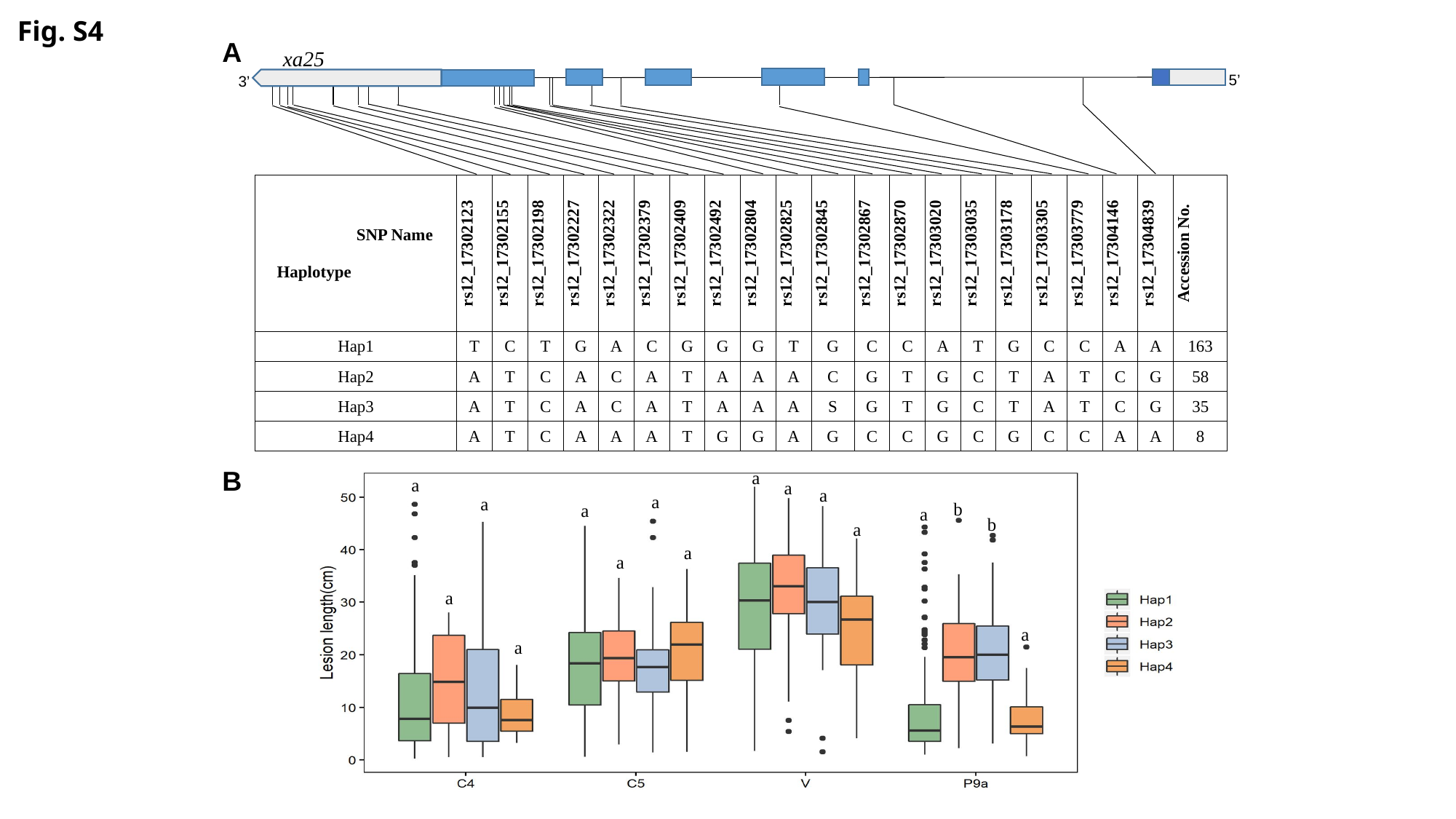

Fig. S4
A
xa25
5’
3’
| SNP Name Haplotype | rs12\_17302123 | rs12\_17302155 | rs12\_17302198 | rs12\_17302227 | rs12\_17302322 | rs12\_17302379 | rs12\_17302409 | rs12\_17302492 | rs12\_17302804 | rs12\_17302825 | rs12\_17302845 | rs12\_17302867 | rs12\_17302870 | rs12\_17303020 | rs12\_17303035 | rs12\_17303178 | rs12\_17303305 | rs12\_17303779 | rs12\_17304146 | rs12\_17304839 | Accession No. |
| --- | --- | --- | --- | --- | --- | --- | --- | --- | --- | --- | --- | --- | --- | --- | --- | --- | --- | --- | --- | --- | --- |
| Hap1 | T | C | T | G | A | C | G | G | G | T | G | C | C | A | T | G | C | C | A | A | 163 |
| Hap2 | A | T | C | A | C | A | T | A | A | A | C | G | T | G | C | T | A | T | C | G | 58 |
| Hap3 | A | T | C | A | C | A | T | A | A | A | S | G | T | G | C | T | A | T | C | G | 35 |
| Hap4 | A | T | C | A | A | A | T | G | G | A | G | C | C | G | C | G | C | C | A | A | 8 |
B
a
a
a
a
a
a
b
a
a
b
a
a
a
a
a
a

## Slide 5
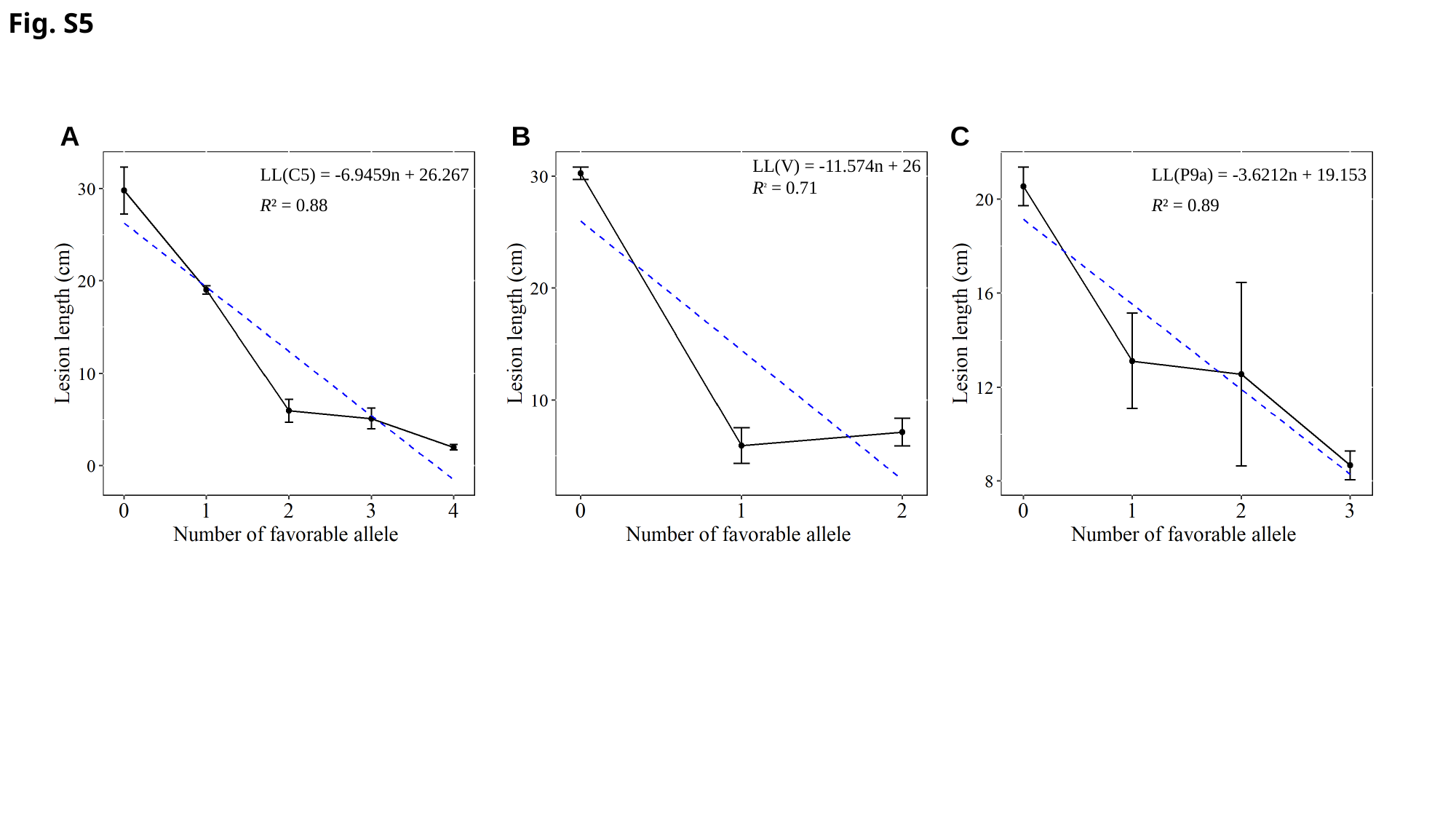

Fig. S5
A
B
C
LL(C5) = -6.9459n + 26.267
R² = 0.88
LL(V) = -11.574n + 26
R² = 0.71
LL(P9a) = -3.6212n + 19.153
R² = 0.89

## Slide 6
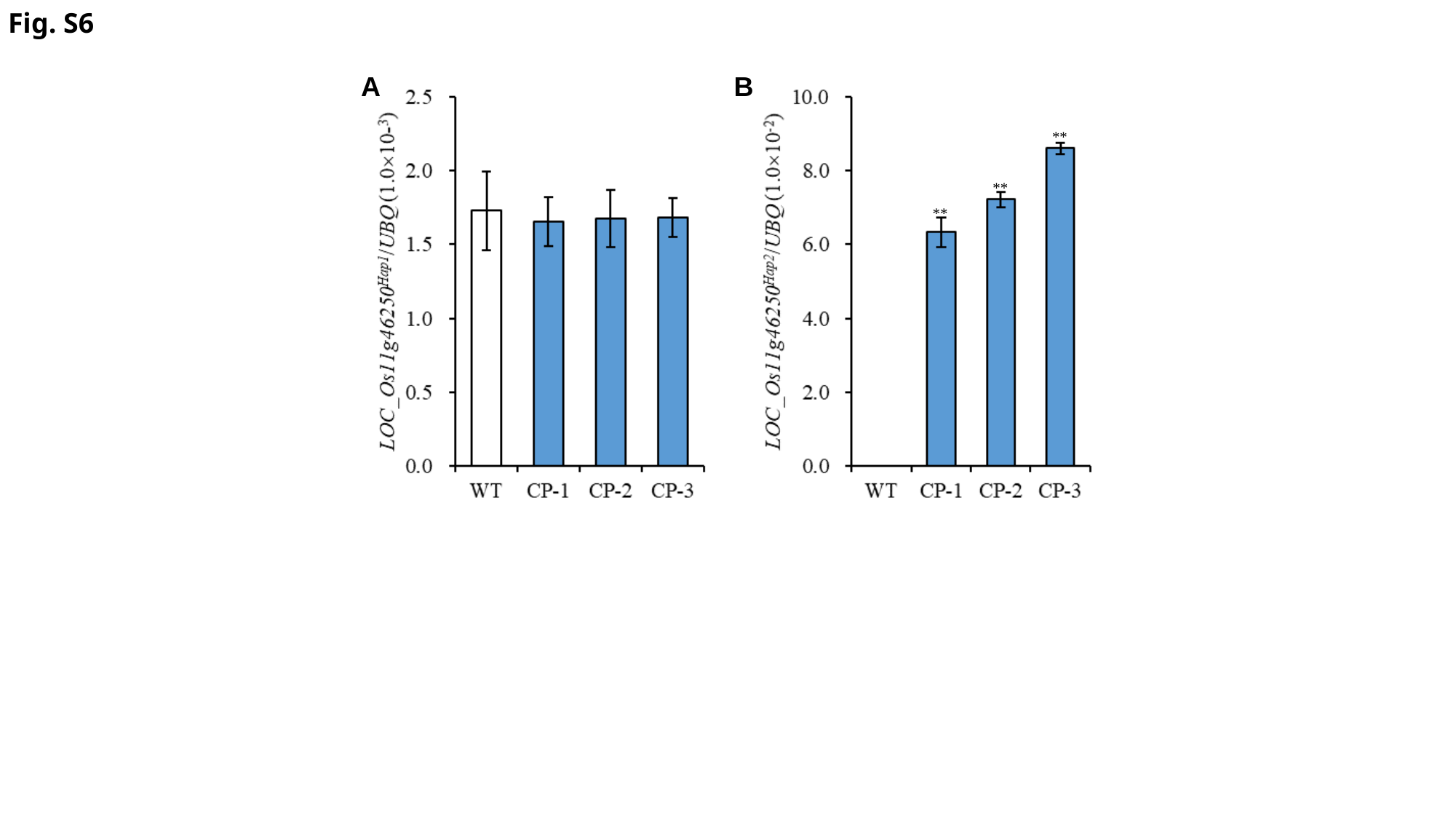

Fig. S6
A
B
**
**
**

## Slide 7
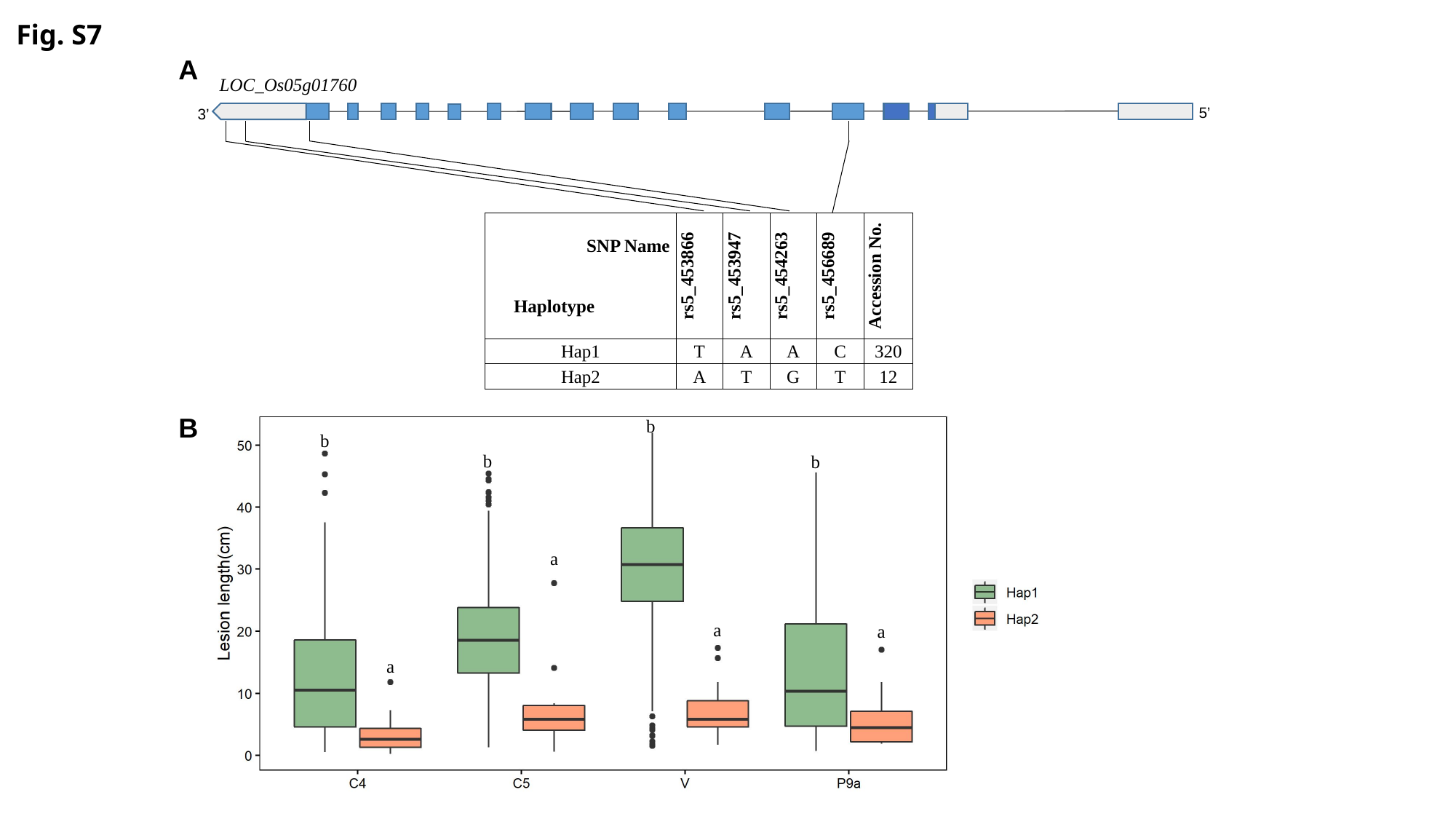

Fig. S7
A
LOC_Os05g01760
5’
3’
| SNP Name Haplotype | rs5\_453866 | rs5\_453947 | rs5\_454263 | rs5\_456689 | Accession No. |
| --- | --- | --- | --- | --- | --- |
| Hap1 | T | A | A | C | 320 |
| Hap2 | A | T | G | T | 12 |
B
b
b
b
b
a
a
a
a
